# Impact of Medical Blog Reading and Information Presentation on Readers’ Preventative Health Intentions: Mixed Methods, Multistudy Investigation

**DOI:** 10.2196/23210

**Published:** 2021-12-22

**Authors:** Kimberly A Taylor, William F Humphrey Jr

**Affiliations:** 1 Department of Marketing & Logistics College of Business Florida International University Miami, FL United States

**Keywords:** health blogs, patient blogs, preventative care, cancer, caregivers, perceived risk

## Abstract

**Background:**

Medical blogs have become valuable information sources for patients and caregivers. Most research has focused on patients’ creation of blogs as therapy. But we know less about how these blogs affect their readers and what format of information influences readers to take preventative health actions.

**Objective:**

This study aimed to identify how reading patient medical blogs influences readers’ perceived health risk and their intentions to engage in preventative health actions. Further, we aimed to examine the format of the medical blog and the reader’s response.

**Methods:**

We surveyed 99 university participants and a general-population, online panel of 167 participants. Both studies randomly assigned participants to conditions and measured blog evaluation, intentions for preventative health action, and evaluation of health risk and beliefs, and allowed open-ended comments. The second study used a different sample and added a control condition. A third study used a convenience sample of blog readers to evaluate the link between reading medical blogs and taking preventative health action.

**Results:**

Across 3 studies, participants indicated a desire to take future preventative health action after reading patient blogs. Studies 1 and 2 used experimental scenario-based designs, while Study 3 employed a qualitative design with real blog readers. The 2 experimental studies showed that the type of blog impacted intentions to engage in future preventative health actions (Study 1: *F*_2,96_=6.08, *P*=.003; Study 2: *F*_3,166_=2.59, *P*=.06), with a statistical blog being most effective in both studies and a personal narrative blog showing similar effectiveness in Study 2, contrary to some prior research. The readers’ perceptions of their own health risk did not impact the relationship between the blog type and health intentions. In contrast, in one study, participants’ judgments about the barriers they might face to accessing care improved the fit of the model (*F*_2,95_=13.57, *P*<.001). In Study 3’s sample of medical blog readers, 53% (24/45) reported taking preventative health action after reading a health blog, including performing a self-check, asking a doctor about their health risk, or requesting a screening test. Additionally, these readers expressed that they read the blogs to follow the author (patient) and to learn general health information. All studies demonstrated the blogs were somewhat sad and emotional but also informative and well-written. They noted that the blogs made them appreciate life more and motivated them to consider taking some action regarding their health.

**Conclusions:**

Reading patient blogs influences intentions to take future health actions. However, blog formats show different efficacy, and the readers’ disease risk perceptions do not. Physicians, medical practitioners, and health organizations may find it useful to curate or promote selected medical blogs to influence patient behavior.

## Introduction

### Background

Patients and caregivers rely on online blogs, social media posts, or online health communities to share information about their illness, treatment paths, or feelings about their condition [1]. Some share with a small circle of friends and family, while others make their blog posts public and shareable with a worldwide audience. Many studies have shown the therapeutic power that writing has for patients [2,3]. Some cancer organizations now even offer writing classes and activities for survivors [4], and many health care providers are writing blogs as well [5,6]. Comparatively less research has focused on the blog readers, however.

Over the years, the web has become more interactive and “participatory” with many peer-to-peer communication forms, such as social media, blogs, and wikis, making up much of what we do online [7]. Softwarefindr estimated there were over 505 million blogs online [8] in 2018. As of 2020, internet users create new posts every 0.5 seconds, while 77% of them say they read blogs regularly [9]. Though lifestyle topics are among the most popular categories [10], personal health and illness-related blogs have proliferated as many people go to the internet as their first source of health-related information [11].

As the Pew Internet Project explains, “peer-to-peer health care acknowledges that patients and caregivers know things—about themselves, about each other, about treatments—and they want to share what they know to help other people.” We have a natural tendency to want to both seek and share information about our health, and technological advances have made this easier than ever [12]. The internet allows community interactions between individuals, including patients and caregivers, and it can be a resource for individuals to discover health information. Further, it has facilitated access to health information and emotional support [13,14]. Social media conversations also have proven effective in improving patient knowledge of their conditions and reducing anxiety [14], but research has demonstrated that individuals are more likely to consume social content instead of creating it [15]. In fact, while the internet has become more participatory, a relatively smaller group of “power users” contribute more than the average user [15].

Researchers studying cancer patient bloggers found that writing their own and reading other patients’ stories affected these patients’ perceptions of their illnesses and prognoses [16]. These cancer patients kept blogs to be remembered after their death, to release negative emotions and frustrations, and to share information about their experience with others, especially other patients. Additional research found that patients explore other patients’ experiences, find community and belonging, and gain a sense of hope by reading and following survivor stories on social media [17-19]. A study of 5 women experiencing depression reported similar results, finding that the blogs helped the writers stave off feelings of seclusion, find community, and form bonds with readers [20]. Moreover, the author coined the term “narrative sandbox” to refer to these blogs as a “protected virtual space that allows bloggers to temporarily and experimentally add or remove different sections from their narrative” [20]. As such, these narratives are dynamic and changeable, with the blog readers becoming “active participants in the writing and rewriting of bloggers’ depression narratives” [20].

Caregivers—usually parents of ill children, adult children caring for aging parents, or the patient’s spouse—write many health blogs as well, and studies of cancer caregiving report the key motivations: “to report, explain, express, reflect, archive and advocate” [21]. Thus, these parent blogs provide information to readers by reporting events and explanations of medical terms while also expressing feelings, emotions, and reflections about events. A study of blogs by caregivers of dementia patients found similar themes, including social support and engagement, gathering and sharing information, reminiscing and building legacies, and altruism [22]. Just as the parents of children with cancer often have to advocate for their children, the family members of patients with dementia often feel drawn to activism and want to advocate for others. Some researchers argue that blogs about the end of life are understudied. In her analysis of 3 late-stage cancer blogs, Andersson [23] posits that the blogs provide useful language around illness, death, and dying, which in turn produces emotional responses in the reader. However, the paper also recognized the deficiencies in our language and feelings of powerlessness. The paper noted that the blogs often feature narratives of struggle and fighting the disease, positive thinking, and even magical thinking, but little direct discussion of death. This lack of adequate language for discussing death creates a meaningful bond between the writer and the reader in their “shared ineffability” or an inability to properly express their thoughts and feelings about death.

### Blog Readers and Health Beliefs

We have seen that blogs about a treatment plan can enhance patient-provider relationships [24], and participation in online groups can help empower patients and counteract isolation [25]. Yet, researchers know comparatively less about the influence of these blogs on their readers. Much of the literature on reading medical blogs refers to medical students as the readers and how these blogs enhance medical education [26,27]. Although that is indeed a worthwhile goal, medical students are not the target of the present research.

One survey of cancer blog users identified 3 different clusters [28] that varied by their motivations for reading blogs, and one segment had the most behavioral change (seeking changes in care) due to the blogs. However, 59.29% of their sample were cancer patients, 31.86% were family or friends of cancer patients, and the remaining 6.19% were medical professionals, and this study did not explicitly differentiate between the characteristics of the blog content or between blog users who were the writers and those who were readers.

A study that explicitly studied blog readers found 4 reasons for reading blogs and linked these to 3 behavioral outcomes [29]. The 4 motivations for reading blogs included “affective exchange, information search, entertainment, and getting on the bandwagon,” while outcomes included changing readers’ opinions, reader-writer interaction, and spread of word-of-mouth communications to others. Thus, given that reading blogs can change one’s opinions and motivate some actions, it is reasonable to conclude that readers might seek to monitor their health or take preventative action upon reading an illness blog.

Some characteristics of an illness blog may produce greater behavioral change than others. According to the Stanford Encyclopedia of Philosophy, Aristotle presented 3 means to persuasion: the character or credibility of the speaker (*ethos*), the emotional response of the listener (*pathos*), or the argument itself (*logos*) [30]. In addition, Cialdini [31] proposed that people employ decisional heuristics, or shortcuts, to deal with the volume of information they encounter. One such heuristic is similarity, in which people trust and believe those they deem to be like themselves [32]. Past research also has associated social proof, another persuasion tool in which we follow what we see others doing, with persuasion and conforming to established norms [33]. Similarly, other persuasion tools of commitment and consistency by Resnik and Cialdini [33] have also been associated with compliance in behavior.

This research is interested in patient blogs rather than those written by medical professionals. Thus, we did not investigate the writer’s credibility (ethos) due to specialized knowledge or expertise. Instead, the study considers the blog’s emotional response (pathos) through a personal narrative, which may demonstrate similarity or social proof to the reader, and objective data in the form of cancer statistics (logos), which may be an indication of disease risk.

Past research found that personal stories (narratives) increased both the perceived risk of infection with the hepatitis B virus and the intention to vaccinate more than statistical evidence [34]. This research suggested that personal narratives are less prone to counterarguing or discounting by the reader, as messages inconsistent with one’s prior beliefs have been shown to be [35]. Thus, the researchers proposed: “narratives are hence expected to be superior in conveying personal health risks than statistical evidence” [35]. Similar work found statistical data were less persuasive in an alcohol education message [36], while other research found anticipated emotions more strongly predicted influenza vaccination than perceived risk [37]. The presentation of objective information caused people to rely on a peripheral cue—the expertise of the source—“so that an expert communicator induced greater persuasion than did a source with lower expertise” [38]. Readers would not be likely to consider a patient to be an expert source, and thus, perhaps, patient blogs might be less persuasive when written with statistical data than when written as a personal story.

### Objective

While researchers have explored patient use of writing, we know less about the readers of these writings, and these studies aimed to fill this gap. The research’s primary objective was to determine whether medical blog readers intend to take some health action specifically after reading a health blog (objective 1).

Specifically, we posited that blog type will significantly affect intentions to take some personal health action, with a *personal narrative* eliciting higher intentions than a blog with *statistical data*. Thus, the secondary objective was to determine specific characteristics of the most likely blogs to produce these health changes (objective 2). The final objective was to determine whether the blog reader’s perceived health risk will mediate the effect of blog type on behavioral intentions (objective 3). That is, do readers believe themselves to be at higher risk after reading a health blog and then take some preventative action? To explore the 3 objectives, we employed a combination of quantitative and qualitative methods across 3 studies.

## Methods

### Study Design

All research was conducted in the United States using Qualtrics XM survey software to randomize treatment conditions for Studies 1 and 2. Study 3 was a qualitative survey with no treatment conditions.

#### Studies 1 and 2

The first study was a single-factor design with 3 levels of medical blog types. This study presented participants with 1 of 3 cancer blog excerpts—a personal narrative (story), a blog with statistical data about disease prevalence, and a more general cancer blog. The second study replicated those 3 conditions with a different sample population and added a control condition (with no blog excerpt).

#### Study 3

A third study of real blog readers provided some external validity and assessed actual preventative health actions taken (rather than hypothetical, intended actions) after reading a patient blog. This study utilized a qualitative survey with no intervention or experimental conditions.

### Participants

#### Study 1

The participants were 99 students in an academic research subject pool at a large southeastern public university. The student participants received a few extra credit points in a college class in exchange for participating in the study. Potential participants saw a brief description of the study posted using SONA research panel software, where they could decide which studies they wished to complete. The completion rate was 97.1% (99/102) of the sample, while the remaining 2.9% (3/102) abandoned the online survey without answering any questions and thus were dropped from the final sample. The sample had an average age of 24.5 years and was 53% (53/99) female.

#### Study 2

Study 2 used a sample from the Amazon Mechanical Turk (MTurk) panel who were over 18 years old. Amazon workers have their choice of which studies they wish to complete and when. Participants included 167 Amazon master MTurk workers (past participants who have provided quality responses to other researchers) who received a US $2 payment for participation. An additional 22 participants began the survey and read the online consent form but answered no other questions; they were thus dropped from the final dataset, resulting in an 88.4% (167/189) participation rate. Respondents were 56.4% (94/167) female with an average age of 44.8 years.

#### Study 3

Participants were a convenience sample of actual medical blog readers recruited through posts to the authors’ social media, which were then reposted or forwarded in a snowball sample. Although 59 participants began the survey, 8 participants said they had not read any medical blogs, and 3 participants were not sure or did not respond to the question. An additional 3 participants responded that they had read a medical blog but did not answer any further questions. Thus, the final sample contained responses from 45 participants. Table 1 summarizes the sampling, recruitment, and analysis methods employed in the 3 studies.

**Table 1:** Summary of participants in the 3 studies that focused on blog intention investigations.

Characteristics		Study 1 (n=99)	Study 2 (n=167)	Study 3 (n=45)
Type of sample		University research panel	Amazon MTurk master respondent panel	Convenience and snowball sample of medical blog readers
Average age (years), mean (range)		24.5 (18-46)	44.8 (21-76)	Not collected
Location		Southwestern US research university	United States	United States
Research tools		Qualtrics XM Survey Software	Qualtrics XM Survey Software	Qualtrics XM Survey Software
Development of tools		Adaptation of existing scales and creation of blog posts	Adaptation of existing scales and creation of blog posts	Blog evaluation scales repeated from studies 1 & 2 and additional questions
Prior relationship with researcher		Primarily no (4 of the 99 were enrolled in the researcher’s course)	No	Some—convenience snowball sample
Blinded		No—identifying information collected in a separate file from participant response data, per institution IRB^a^ guidelines	Yes	Yes
**Statistical analysis**				
	Quantitative data	One-way ANOVA^b^ (SPSS)	One-way ANOVA, stepwise regression (SPSS)	Simple means and % reported
	Qualitative data	Hand coding of open-ended responses	Hand coding of open-ended responses	No coding—qualitative responses reviewed and summarized
Female, n (%)		53 (53.3)	94 (56.4)	Not collected
Education		Current undergraduate students	Not provided by panel	Not collected
Recruitment		University research panel software: Sona Systems	Posted on Amazon MTurk^c^ open studies to eligible panelists	Recruitment via social media and snowball sample
Percentage of original sample that completed the study, %		97.1^d^	88.4^e^	76.3^f^

^a^IRB: institutional review board.

^b^ANOVA: analysis of variance.

^c^MTurk: Mechanical Turk.

^d^N=102.

^e^N=189.

^f^N=59.

### Intervention and Instruments

#### Study 1

Survey instruments were developed primarily by adapting existing materials and scales. The researchers adapted the blog posts used in Studies 1 and 2 from a publicly available, widely shared blog post about someone with skin cancer. This public blog excerpt was adapted and revised to create the 3 conditions (cancer statistics added to create that condition, for example). All blog posts were similar in total word length and readability. The Qualtrics survey software randomly assigned participants to 1 of 3 experimental conditions (for Study 1), which differed only in which blog post was presented to participants. The 3 blog types were a personal narrative (focused exclusively on a patient’s personal story), a general cancer story, and a third statistics condition (that incorporated data and statistics about melanoma, rather than a personal story). All sample blog posts featured the same photo of a young woman and were approximately the same length.

After first reading the informed consent form and consenting to participate, subjects saw the sample blog post and answered an open-ended question asking their thoughts about the blog. This open-ended question was followed by an evaluation of the blog itself in terms of readability, informativeness, and interest on 7-point bipolar scales (all specific scale items and instruments are included in Multimedia Appendix 1).

Next, participants indicated how likely they were to engage in several preventative health actions (see a doctor for a skin check, monitor your skin yourself for any changes, use sunscreen daily, use sunscreen when going to the beach, or ask a doctor about cancer risk) on 7-point scales (from 1 = not at all likely to 7 = extremely likely). Then, respondents completed health beliefs model scales [39]; scales were reworded only to relate to skin cancer or skin checks rather than vaccination. These scales comprised measures of perceived barriers to seeking care, perceived benefits of skin checks, perceived susceptibility to skin cancer, and perceived severity of the effects of skin cancer. Although not intended to be predictive, we included these scales to explore potential relationships between constructs.

Finally, participants completed locus of control (loc) scales measuring internal, external-other (in which powerful others control events), and external-chance (in which events are due to fate or luck) loci of control [39]. The survey concluded with a few personal health questions (whether they used sunscreen and how often, whether they have ever had a skin check with a dermatologist, and whether they had a prior diagnosis of skin cancer). On average, participants spent 652.85 seconds (nearly 11 minutes) on the survey.

#### Study 2

Study 2 was conducted to replicate the significant effects found in Study 1 with a larger, general adult (nonstudent) sample population. Moreover, we added a control condition in which participants responded to the health intention, health beliefs, and locus of control measures but were not shown any blog post. Thus, for this study, the Qualtrics software randomly assigned participants either to 1 of the 3 blog-type conditions or to the no blog condition.

All study manipulations and measures were identical to those of Study 1, except for the addition of the control condition. In the control condition, instead of presenting a blog sample, we asked participants where they usually get their health information and their evaluations of that source. This design kept the survey to a similar length and provided participants a task to complete before answering the health intention measures. On average, respondents spent 618.17 seconds (10.3 minutes) on the survey.

#### Study 3

The first 2 studies were hypothetical, scenario-based designs. Thus, we also conducted a concise survey of real blog readers to explore our research objectives and assess the effect of medical blogs on actual preventative behaviors (rather than intentions). After asking respondents to think about a medical blog that they had read or followed, the survey asked who wrote the blog they read, whether they took any personal health-related actions after reading the blog, and if so, what actions. Next, the survey asked their reasons for reading the blog, what they liked or did not like about reading the blog, and for an evaluation of the blog writing itself. The researchers developed all measures for this study. These respondents completed the 7-question survey in an average of 212.9 seconds (3.55 minutes).

### Statistical Analysis

We analyzed the quantitative scale data in Studies 1 and 2 with one-way analyses of variance (ANOVAs) and a simple regression model using SPSS version 26. Study 3 did not include an intervention; thus, the only quantitative data reported for that study were descriptive means and percentages.

The qualitative data from Studies 1 and 2 were analyzed through a coding process. First, researchers developed a list of codes for the open-ended, written evaluations of the blog posts based upon prior literature and an initial review of a sample of responses. All thought listings were downloaded to a spreadsheet and separated by respondent. Next, 2 coders, blind to both experimental condition and research objectives, independently coded all responses manually in the spreadsheet. Across the data from Studies 1 and 2, there was nearly 70% agreement (69.3%) between the 2 coders with a κ value of 0.63 [40]. This was deemed a sufficiently strong level of agreement, and a third coder resolved discrepancies.

### Ethics

All studies were approved by the Social and Behavioral Sciences Institutional Review Board (IRB) at a large, public university in the southeastern United States (IRB 20-0088). Participants for all studies completed an online consent form prior to beginning the study and were free to omit any questions or leave the study at any time.

Study 1 was not blinded, as participant information was required for awarding the compensation (of the extra class credit). However, in accordance with the university’s IRB guidelines, the identifying information was saved to a separate file and not connected with the experimental responses. Studies 2 and 3 were blinded. Amazon MTurk (used for Study 2) provides only a user ID, and participant identities are not revealed to researchers. The final qualitative survey (Study 3) used Qualtrics anonymous response settings and did not capture any identifying information.

## Results

### Study 1

The blog excerpts were rated overall as being easy to read (mean 5.84, SD 1.61), easy to understand (mean 6.29, SD 1.07), well-written (mean 6.02, SD 1.34), informative (mean 5.64, SD 1.31), emotional (mean 6.16, SD 1.07), and interesting (mean 5.82, SD 1.37). Importantly, these evaluations did not differ between the 3 blog conditions (all *F*_2,98_ values <1.3, *P*>.30 for one-way ANOVAs). That is, the different blog types were judged to be equally informative, easy to read, and emotional. Thus, any significant effects of blog type on the health intentions could not be attributed to these characteristics. Table 2 summarizes the individual means for the evaluation of the blog posts, as well as the behavioral intentions.

**Table 2:** Study 1 blog evaluations and behavioral intentions.

Characteristics		Blog type			Overall (N=99), mean (SD)
		Personal (n=34), mean (SD)	General (n=38), mean (SD)	Statistics (n=27), mean (SD)	
**Blog evaluation**					
	Easy to read	5.76 (1.58)	5.92 (1.60)	5.81 (1.71)	5.84 (1.61)
	Easy to understand	6.32 (1.01)	6.18 (1.21)	6.41 (0.97)	6.29 (1.07)
	Well-written	6.06 (1.41)	5.97 (1.33)	6.04 (1.32)	6.02 (1.34)
	Informative	5.50 (1.33)	5.68 (1.25)	5.74 (1.16)	5.64 (1.31)
	Emotional	6.26 (1.05)	6.26 (1.01)	5.89 (1.16)	6.16 (1.07)
	Interesting	5.59 (1.44)	6.00 (1.21)	5.85 (1.49)	5.82 (1.37)
**Behavioral intention**					
	Skin check by a doctor	4.26 (1.83)	4.55 (1.75)	5.04 (1.85)	4.59 (1.81)
	Skin self-check	5.29 (1.43)	5.71 (1.43)	6.30 (0.869)	5.73 (1.35)
	Sunscreen daily	4.15 (2.05)	4.47 (2.19)	5.30 (2.15)	4.59 (2.16)
	Sunscreen at beach	5.85 (1.87)	6.18 (1.45)	6.70 (0.669)	6.21 (1.49)
	Asked a doctor about skin cancer risk	4.58 (1.82)	4.84 (1.94)	6.11 (0.974)	5.10 (1.79)

Next, we examined whether blog type affected the readers’ health intentions. First, the set of health intention measures proved to be reliable as one scale (Cronbach α=.79), with an overall mean of 26.2 (SD 6.4). The effect of blog type on health intentions surrounding skin cancer prevention was significant (*F*_2,96_=6.08, *P*=.003). However, the direction was not as predicted, as both patient blog types (personal: mean 23.97, SD 1.26; general: mean 25.76, SD 6.10) elicited lower intentions to take preventative health action than did the blog that presented statistics without a personal story (mean 29.44, SD 4.54; *P*=.001, *P*=.02, respectively). Table 2 summarizes the means of the individual health intention measures for all conditions.

On the individual health intention measures, we observed significant differences between blog conditions only for self-monitoring of one’s skin (*F*_2,98_=4.47, *P*=.01) and asking a doctor about one’s cancer risk (*F*_2,97_=6.9, *P*=.002). On both measures, the blog with statistics led to greater preventative health intentions than either of the other 2 blog conditions (personal narrative and general cancer–related.) The 2 measures about using sunscreen demonstrated *P* values of .11 and .08, respectively, while the first measure about seeing a doctor for a skin check did not differ between blog conditions (*P*=.26).

The third research proposition was that blog type would influence health beliefs. First, we assessed the reliability of all health beliefs subscales, including the perceived barriers to accessing health care (Cronbach α=.63), perceived susceptibility (Cronbach α=.68), and perceived severity subscales (Cronbach α=.69; the perceived benefit of getting a skin check was a single-item measure, not a multi-item scale). However, the reliability of the perceived barriers scale was higher (Cronbach α=.65) without the second item (“a skin check could have unpleasant side effects”), and the reliability of the perceived severity subscale was higher without the first item (“skin cancer may lead to serious health problems”; α=.712 without that item). Thus, the final reported scales did not include those items. Moreover, the external-others locus of control scale was used in full (α=.78), while both the internal loc and external-chance loc scales had greater reliability if some items were dropped (α_s_=.78 for resulting scales; internal loc removed the first 2 items, and chance loc removed the third item); thus, the reduced scale means are reported in the following sections. Table 3 summarizes the health belief subscales and locus of control scales (described in subsequent sections), and all scale items are listed in Multimedia Appendix 1.

**Table 3:** Study 1 locus of control.

Locus of control (LOC)	Blog type			Overall, mean (SD)	Number of items
	Personal, mean (SD)	General, mean (SD)	Statistics, mean (SD)		
Perceived barriers	14.06 (5.18)	12.46 (3.97)	11.08 (4.12)	12.62 (4.57)	4
Perceived benefits	5.19 (1.38)	5.32 (1.44)	5.85 (1.32)	5.42 (1.398)	1
Perceived susceptibility	8.75 (3.99)	8.76 (3.23)	9.96 (4.38)	9.09 (3.83)	3
Perceived severity	17.24 (5.84)	18.53 (5.49)	20.00 (6.08)	18.50 (5.81)	5
Internal LOC	18.70 (4.77)	18.84 (4.68)	18.81 (4.21)	18.79 (4.54)	4
Powerful others LOC	24.39 (7.21)	24.79 (6.64)	25.52 (5.08)	24.86 (6.41)	6
Chance LOC	13.91 (6.23)	15.03 (5.88)	14.52 (4.53)	14.51 (5.63)	5

Neither the perceived susceptibility to skin cancer (*F*_2,96_ =.965 *P*=.38) nor the perceived severity subscales (*F*_2,97_=1.7, *P*=.19) varied by blog condition. The perceived benefits of skin checks also did not differ by condition. However, perceived barriers to screening did significantly differ by blog condition (*F*_2,96_=3.373, *P*=.04), with the personal narrative showing greater barriers than the statistics blog. We did not predict a priori any effects of blog condition on internal, powerful others or chance locus of control, and none were found (all *F* values <1, all *P*>.70).

Given that the perceived barriers to accessing care differed by blog type, we incorporated that scale alone into a regression model. A simple linear regression analysis regressing both blog type and perceived barriers to care onto the health intentions scale yielded an overall model that was significant (*F*_2,95_=13.57, *P*<.001), as well as both variables that were significant (β_blog_=.227, *t*_92_=2.38, *P*=.02; β_perbarrier_=–.36, *t*_92_=–3.74, *P*<.001). Thus, adding the perceived barriers to care does not eliminate the effect of blog type on health intentions. Moreover, retaining the blog type in the regression model improved the model *R*^2^ from .179 to .226 (Table 4).

**Table 4:** Regression analysis results.

Model factor	Estimate	SE	95% CI LL^a^	95% CI UL^b^	*P* value
(Constant)	10.951	2.679	5.630	16.271	<.001
PerBarScale	0.510	0.136	0.240	0.781	<.001
Blog version	–1.859	0.780	–3.409	–0.310	.02

^a^LL: lower limit.

^b^UL: upper limit.

Next, we turned our attention to the analysis of the open-ended question about the blog. The codes used for this qualitative analysis are shown in Table 5, along with sample responses and the number of responses coded into each category. The most-reported codes for the Study 1 sample were sadness/sympathy/empathy (code 1), makes one appreciate life more (code 7), informative (code 4), well-written (code 5), and makes one think of someone with cancer (code 8).

**Table 5:** Coding of open-ended responses and sample respondent statements in Study 1.

Open-ended question code category	Sample statements	Number of responses
1. Sadness/sympathy/empathy	I feel saddened by the writer’s situation and an urge to help by any means necessary.	17
2. Feel guilty (eg, to be alive and well when others are not)	I feel guilty that I do not appreciate my life enough.	1
3. Feel worried/concerned for oneself	I feel nervous when I think about this sort of thing happening.	0
4. Informative	I think that it is informative about the feelings of people with cancer, which is something that you do not hear about often.	11
5. Well-written	I think that Natalie is an excellent writer who is expressing her true feelings about having cancer.	9
6. Motivates behavior (eg, will make an appointment, do a skin check)	After reading this, I really felt like I want to go and do my annuals, which is pending.	2
7. Makes you appreciate life more	That life is a gift. And we need to leave it with meaning and love what we do. Be thankful.	13
8. Thought of someone with cancer	I’m thinking of all of the people that I know who have had cancer. I have lost so many family members to cancer.	9
9. Did not want to read/would not read	I immediately wanted to close the page when I saw the topic. I feel sick to my stomach. This is not a topic I would ever want to read about.	4
10. No new information/heard it all before	There are many blogs like that, and I have heard many similar ones.	2

The distribution of codes across the 3 blog versions was also significantly different (*X*^2^_16,68_=28.54, *P*=.03). Both personal blog types elicited great feelings of sadness and sympathy (code 1), with the personal narrative blog also being considered well-written (code 5) and the general blog making readers appreciate their own life more (code 7). Participants most often cited the statistics blog as being informative (code 4). Notably, 3% (2 out of the 68 who provided a response to the open-ended question) stated, without prompting, that the blog made them intend to take some personal medical action.

### Study 2

In this sample, the blog excerpts were again rated overall as being easy to read (mean 6.28, SD 1.17), easy to understand (mean 6.65, SD 0.724), well-written (mean 6.10, SD 1.28), informative (mean 5.47, SD 1.43), emotional (mean 5.91, SD 1.26), and interesting (mean 5.64, SD 1.62). There were few differences in these evaluations across the 3 blog types, except for how understandable (*F*_2,123_=3.45, *P*=.04) and emotional (*F*_2,123_=3.40, *P*=.04) the blogs were. The blog with the very personal story was rated more understandable than the general blog (*P*=.01), and the personal story was rated more emotional than the statistics-focused version (*P*=.01). Table 6 summarizes the evaluations of the blog posts.

**Table 6:** Study 2 blog evaluations.

Characteristic	Blog type			Overall (N=167), mean (SD)
	Personal (n=42), mean (SD)	General (n=41), mean (SD)	Statistics (n=41), mean (SD)	
Easy to read	6.14 (1.42)	6.12 (1.21)	6.59 (0.706)	6.28 (1.17)
Easy to understand	6.81 (0.505)	6.41 (0.974)	6.71 (0.559)	6.65 (0.724)
Well-written	6.26 (1.27)	5.85 (1.30)	6.17 (1.26)	6.10 (1.28)
Informative	5.38 (1.58)	5.24 (1.46)	5.78 (1.22)	5.47 (1.43)
Emotional	6.19 (1.37)	6.02 (1.24)	5.51 (1.08)	5.91 (1.26)
Interesting	5.76 (1.57)	5.56 (1.67)	5.59 (1.63)	5.64 (1.62)

Next, we turned our attention to the primary research objectives—does reading a personal illness blog affect the reader’s own health intentions (objective 1)? Specifically, is there an effect of the blog type on these health intentions (objective 2)? First, the set of health intention measures proved to be reliable as a scale (Cronbach α=.80), with an overall mean of 24.48 (SD 6.94). The experimental condition’s effect on health intentions was not significant in the overall ANOVA (*F*_3,166_=2.59, *P*=.06). However, looking at the planned comparisons revealed that all blog conditions led to higher health intentions than the control (no blog) condition, but there were no significant differences between the 3 experimental blog conditions. Specifically, the personal (mean 25.21, SD 6.64; *P*=.03) and statistical (mean 25.95, SD 6.81; *P*=.01) blog versions led to higher overall health intentions than did the no blog control (mean 22.02, SD 7.27) condition. The general blog (mean 24.49, SD 6.84) condition did not significantly differ from either the control or the other blog conditions.

One-way ANOVAs conducted on the individual health intention measures showed a significant difference existed only for daily sunscreen use (*F*_3,166_=4.39, *P*=.005), with the only other noteworthy comparison observed for getting a skin check by a doctor (*F*_3,166_=2.06, *P*=.11). Post hoc tests showed that, for daily sunscreen usage, all blog conditions indicated greater intentions than the no blog condition (*P*=.002, *P=*.004, and *P*=.005, respectively), while both the personal story and statistics blog versions led to greater intentions to get one’s skin checked by a doctor than the no blog condition (*P*=.04 and *P*=.03, respectively). Table 7 summarizes the health intention measures by experimental condition.

**Table 7:** Study 2 behavioral intentions.

Characteristic	Control (no blog), mean (SD)	Blog type			Overall (N=167), mean (SD)
		Personal (n=42), mean (SD)	General (n=41), mean (SD)	Statistics (n=41), mean (SD)	
Skin check by a doctor	3.42 (2.04)	4.33 (2.09)	3.88 (2.05)	4.39 (2.02)	4.00 (2.07)
Skin self-check	5.65 (1.36)	5.74 (1.74)	5.71 (1.33)	5.95 (1.52)	5.76 (1.49)
Sunscreen daily	3.28 (2.33)	4.69 (2.12)	4.61 (2.02)	4.56 (1.86)	4.28 (2.16)
Sunscreen at beach	5.95 (1.75)	6.33 (1.41)	5.93 (1.88)	6.54 (1.31)	6.19 (1.61)
Ask a doctor about skin cancer risk	3.81 (1.92)	4.12 (1.82)	4.37 (1.91)	4.51 (2.03)	4.20 (1.92)

As we did collect demographic data on age and gender in this study, we also evaluated whether either variable affected the behavioral intentions, and they did not. The average response by men (mean 23.91, SD 6.71) on the health intentions scale did not significantly differ from that of the female-identifying respondents (mean 25.11, SD 7.00; *t*_158_=1.1; *P*=.27), nor were there any gender differences on any of the specific items (all *P* values >.13). A regression of age on behavioral intention also was not significant (*F*_2,161_=1.3, *P*=.28).

Respondents in this study also completed the health beliefs and locus of control scales, which we used to consider our third research objective about readers’ perceived risk. Although there is some criticism of how the Cronbach α statistic is used and disagreement as to what constitutes an acceptable reliability, values above .6 or .7 are frequently reported as acceptable [41]. All scales here demonstrated Cronbach α>.62: perceived barriers (Cronbach α=.79), perceived susceptibility (Cronbach α=.63), perceived severity (Cronbach α=.63), internal locus of control (Cronbach α=.77), external-other locus of control (Cronbach α=.82), and external-chance locus of control (Cronbach α=.84). Moreover, there were no differences on any of these scales by experimental condition (all *F*<1.2, *P*>.20). This implies that neither the reading of a blog (versus no blog) nor the different types of blogs affected any of the respondents’ health beliefs or their loci of control. Post hoc comparisons did, however, reveal a significant difference in evaluations of external-chance locus of control between the statistics blog (mean 16, SD 6.24) and the no blog (control) condition (mean 19.12, SD 6.41; *P*=.04); no other differences emerged. Table 8 summarizes these results.

**Table 8:** Study 2 locus of control.

Locus of control (LOC)	Control	Blog type			Overall (N=167), mean (SD)	Number of items
		Personal (n=42), mean (SD)	General (n=41), mean (SD)	Statistics (n=41), mean (SD)		
Perceived barriers	17.1 (6.78)	15.9 (6.07)	16.32 (7.57)	14.68 (5.88)	16.01 (6.61)	5
Perceived benefits	5.52 (1.04)	5.74 (1.15)	5.44 (1.34)	5.88 (1.05)	5.64 (1.16)	1
Perceived susceptibility	9.62 (3.86)	10.10 (3.68)	9.83 (3.73)	10.17 (4.02)	9.93 (3.68)	3
Perceived severity	24.30 (5.64)	24.60 (5.07)	23.55 (5.55)	23.73 (4.98)	24.05 (5.28)	6
Internal LOC	28.88 (4.89)	28.64 (6.00)	29.60 (5.90)	28.93 (5.09)	29.00 (5.46)	6
Powerful others LOC	23.56 (7.11)	24.36 (6.23)	23.29 (7.14)	23.93 (6.63)	23.79 (6.73)	6
Chance LOC	19.12 (6.44)	18.31 (6.92)	17.66 (7.66)	16.00 (6.24)	17.78 (6.87)	6

The number of open-ended thoughts coded into each category for Study 2 are summarized in Table 9. Overall, the most common evaluations were feelings of sadness/sympathy, that the blog was informative, it made them think of someone they know, and that there was no new information presented. Comparing across conditions, we see that the most common responses again varied by blog type (*X*^2^_16,121_=35.49, *P*=.003). For the 2 personal blog versions, the top codes were for expressions of sadness/sympathy/empathy (code 1) and thinking of someone they knew with cancer (code 8). The blog with statistics evoked far different responses, with informative (code 4), well-written (code 5), and motivates behavior (code 6) being the most reported. Across the 3 blog types, 5 responses explicitly expressed worry for oneself, and 8 expressed an intent to be proactive regarding their health (scheduling a doctor’s appointment or getting a skin check, for example.) Thus, without prompting, nearly 11% (13/124, 10.5%) expressed concern about or desire to act regarding their health because of reading one sample blog entry.

**Table 9:** Coding of Study 2 open-ended responses.

Open-ended question code category	Number of responses
1. Sadness/sympathy/empathy	41
2. Feel guilty (eg, to be alive and well when others are not)	0
3. Feel worried/concerned for oneself	5
4. Informative	22
5. Well-written	15
6. Motivates behavior (eg, will make an appointment, do a skin check)	8
7. Makes you appreciate life more	1
8. Thought of someone with cancer	18
9. Did not want to read/would not read	3
10. No new information/heard it all before	18

### Study 3

This nonexperimental study surveyed actual self-described readers of real medical blogs. Most of the blogs read by our study participants were written by patients (27/45, 60%), followed by the patient’s spouse or caregiver (7/45, 16%), a medical professional (6/45, 13%), or the patient’s parent (5/45, 11%). The participants read the blogs for various reasons, chiefly to stay up to date with their friend or family member’s condition (23/45, 51%) and to provide support for their friend (21/45, 47%). Many also read the blogs to gain information about a health condition more generally (11/45, 24%) or to learn information that may be pertinent for their own health (11/45, 24%). Percentages sum to more than 100% because they were able to select more than one reason. They found the blogs easy to read (mean 6.34, SD 1.14), easy to understand (mean 6.25, SD 1.19), well-written (mean 6.13, SD 1.50), informative (mean 6.23, SD 1.38), and interesting (mean 6.24, SD 1.87). Blogs were also considered somewhat emotional (mean 4.97, SD 0.83; all measured on 1 to 7 Likert-type scales).

The central research question for this study (and the paper’s first research objective) was whether readers took any personal health action or changed any behaviors based upon reading the blog, and, indeed, 24 respondents (24/45, 53%) reported doing so. These responses are summarized in Table 10. Of participants who reported taking action, 29% (7/24) scheduled a doctor’s appointment, while others reported requesting a cancer or other health screening (7/24, 29%); performing a self-exam, such as a skin or a breast check (5/24, 21%); or asking a doctor about their own risk (4/24, 17%). Additionally, 54% (13/24) reported making other health changes, including taking supplements, making lifestyle modifications, purchasing cancer insurance, doing additional research, or making donations to disease research.

**Table 10:** Preventative health actions taken by participants in Study 3 (n=45).

Preventative health action taken	Responses, n (%)
Scheduled a doctor’s appointment	7 (16)
Requested cancer or other health screening	7 (16)
Performed a self-exam	5 (11)
Asked a doctor about their own health risk	4 (9)
Other (made other health or lifestyle changes)	13 (29)

Participants cited being able to stay updated, especially without having to impose on their friend or family member for continued updates, as the greatest motivator for reading the medical blog. Additional motivations included learning specific ways they could support the person, feeling connected to the person, and hearing positive news when that was shared. Several also cited that the blog writer was very transparent, real, and open, which they appreciated, or used humor. Comments also corroborated prior findings about the use of medical statistics, with others citing how the writer explained detailed scientific information so that it was understandable for readers, sometimes prompting them to do additional research. Several participants with health challenges also said reading the blogs provided some relief to know that others were going through the same things and helped confirm their own treatment choices. Most said there was nothing they did not like or found difficult about reading the blogs, but 24% (11/45) of participants described the experience as very emotional or sad to read, notably if the person’s condition deteriorated or a treatment was unsuccessful. Those participants appreciated the blog and found it worthwhile to read, despite being sad or emotional at times. Interestingly, a few mentioned that the information was not always correct or that they would take it more as a suggestion for lifestyle modification or a reason to do more research, rather than as medical advice.

## Discussion

### Principal Findings

We conducted 2 scenario-based, experimental studies with different sample populations and 1 qualitative survey of real blog readers to explore the research objectives. All 3 studies confirmed the first research objective, that reading medical blogs was associated with intentions to take preventative health actions, though each study contributed unique findings. Studies 1 and 2 examined the specific characteristics of blogs that led to greater health intentions (objective 2), but neither perceived risk nor severity of skin cancer mediated these effects (objective 3). Study 3 confirmed the first objective with real, rather than intended, behaviors.

### Study 1

This study confirmed that blog type significantly affects intentions to take preventative health action. We predicted that people would be motivated to take health actions upon reading a medical blog and that a highly personalized blog would produce the greatest health intentions. However, we discovered the reverse—the blog with melanoma statistics produced the most significant intention to take preventative health action, higher than either of the other blog conditions, which did not differ. Moreover, the statistics blog also yielded the lowest perceived barriers to accessing care compared with the other blog types. Including both blog type and perceived barriers to accessing care improved the predictive ability of the regression model on health intentions. Thus, blog type significantly affected perceived barriers, which impacted health intentions, but blog type’s direct effect on behavioral intentions retained its significance. We conclude that medical blogs can produce intentions to engage in protective health behaviors, partially through an effect on perceived barriers to care.

### Study 2

Study 2 corroborates and extends the findings of Study 1 by demonstrating that reading a medical blog excerpt led to greater intentions to take preventative health actions compared with a baseline (from a control condition in which participants did not read a blog entry), and this was true for all the blog versions we tested. Further, both a highly personal blog and a statistical blog led to greater personal health intentions than the control condition. In contrast, the more general cancer blog produced a moderate level of health intention that was not statistically different from either the control or the other blog conditions.

However, there were no differences by blog type on any of the health beliefs. Thus, perceived health risk—either susceptibility or severity—again did not mediate the effect of blog type on personal health intentions. Also, blog type and blog reading, in general, did not affect perceptions of either the barriers to or the benefits of skin checks. Interestingly, the statistics-oriented blog did reduce estimates of locus of control attributed to chance.

### Study 3

The final study assessed whether reading medical blogs induced their readers to take any preventative health action in a real-world survey rather than a hypothetical scenario. For a majority of the respondents, it did. While the respondents primarily read the blogs in order to keep up to date with the patient’s condition and provide support, they also found the writing emotional and informative, and many readers took personal health action as a result of reading the blogs. Reading medical blogs inspired people in many ways; they made medical appointments or requested health screenings, conducted additional research on their own, made donations to organizations, or made other lifestyle changes.

### General Discussion

Researchers have long known medical blogs have demonstrable patient benefits, but less was known about the impact on their readers. The top intended health actions across our studies included skin checks and consulting with a physician about cancer risk, both of which are important preventative health actions that can help ensure that a skin cancer is caught early in a more treatable stage [42]. Furthermore, while our first 2 studies measured hypothetical health intentions, much past literature incorporating Ajzen’s Theory of Planned Behavior [43] to health care has found intentions to be predictive of behaviors such as attending health screenings, engaging in healthy eating, or participating in regular physical exercise [44-46]. Thus, the increased intentions are likely to translate into actions, and Study 3 provides some support for that conjecture.

Some of our results also ran counter to predictions. The blog readers in our studies demonstrated a higher propensity to take preventative health actions when blog posts focused on statistics rather than personal stories. Whereas some prior literature showed personal stories to be most persuasive [34], our Study 1 found the statistics-oriented blog to be the most effective, and Study 2 found it to be as persuasive as the personal narrative. Because cancer can be scary and overwhelming to contemplate, there is a natural tendency to believe that cancer patients might have had either genetic or environmental reasons for their cancer diagnosis [47], to distance oneself from the situation and downplay one’s own personal risk. Accordingly, it is possible that a personal story feels more specific to the writer and less relatable to the reader. In the past, people even avoided those with cancer out of fear they might “catch” the disease themselves [48]. This reduced relatability could minimize Cialdini’s [49] similarity persuasion tool of the personal narrative. If one feels that the patient’s story is unique and personal, then the reader may feel sadness and sympathy for the patient (as many of our participants in all 3 studies did) but may not feel motivated to take personal action because they read the story. And in fact, almost 47% of our Study 3 respondents said they did not engage in any preventative health actions after reading the blog.

In retrospect, perhaps the statistical blog increased the social proof aspect of persuasion by illuminating for our study participants that the condition is more widespread than they may have thought [31]. Providing some additional support for this conjecture, Study 1 results showed that the statistics blog reduced perceived barriers to screening compared with the personal story blog. Further, Study 2 showed that the statistics blog reduced the locus of control attributed to chance. Things that occur due to chance or fate, by definition, cannot be prevented by one’s own actions. This effect could play a role in increasing one’s intention to take personal preventative action if the perceived portion of cancer risk due to chance or fate is reduced. Perceived behavioral control—or one’s belief in their ability to perform a behavior—is a central tenet in the Theory of Planned Behavior and has been found to be an important driver of intentions in health care [50]. The prospect of these potential mechanisms deserves further research to clarify our understanding.

We further explored whether the blog post conditions would differ significantly from a control (baseline) condition without any blog post. Indeed, all blog types we studied (general, statistics-focused, and a personal story) demonstrated greater health intentions as compared with the condition with no blog. Specifically, the personal story and statistics-focused blogs were associated with intention to use sunscreen, while all 3 blog types were associated with greater intent to schedule a medical visit for a skin check compared with the control condition. In our real-world follow-up study, we found that the desire to stay up to date on a patient’s condition or find general information motivated survey participants to read medical blogs. As a result of reading these patient posts, more than 53% of respondents indicated that they took a preventative medical action (skin checks, doctor appointments) or other health-related behavior (additional research, insurance purchase, starting supplements). Remarkably, none of the blogs impacted perceived severity of or susceptibility to disease. Thus, perhaps even those readers who may believe they are at low risk might benefit from reading health blogs and be spurred to act.

### Limitations

Limitations of this research relate to natural concessions made in sampling. The first study used a research panel at a major research university, while the second study used a panel from MTurk. Thus, neither sample may be representative of US adults more generally. Nonetheless, both samples included a diverse age range and, despite skewing a bit more female, at least 44% male-identifying respondents. For the sake of parsimony and because we did not have specific research objectives about age or gender, Study 3 did not collect this or any other demographic data. Thus, we cannot compare the results from that study to prior literature that examined these variables. Further, we did not collect educational data for the second or third studies.

Despite limitations inherent with any panel, both experimental studies provided convergent findings and extended our understanding of the efficacy of different blog presentations compared with one another and compared with a baseline without blog content. We designed the final study to understand blog readers’ actual preventative health actions and to increase the robustness of qualitative insights.

The third study used a relatively small convenience sample with snowball sampling to provide insights into real actions resulting from reading patient health blogs. Although qualitative research often precedes quantitative efforts, the first 2 experimental studies’ findings piqued the researchers’ curiosity to explore the research objectives further and gain additional insights with a sample of readers of nonhypothetical medical blogs. Moreover, while we could have included many other variables, we kept the Study 3 survey purposefully short to reduce the burden on participants who received no compensation for participating. This study nonetheless provides an important step in helping to make a connection between blog reading and personal preventative health actions.

Next, this research inquiry looked at specific outcomes related to skin cancer. It is possible that the most influential blog style may differ when investigating other conditions, such as healthy behaviors postbariatric surgery or prevention of the spread of infectious viruses such as COVID-19. We anticipate that a relationship between reading health blogs and intended health actions would exist, but the presentation style and influence of perceived risk might be different. Additionally, these studies are among the first of which we are aware to investigate the impact of reading patient blogs by social media participants. Patients frequently turn to social media and online communities for medical information and advice [51,52]; future research is needed to explore additional predictors and outcomes in this area.

### Conclusions

Blogs from patients undergoing health care treatments have become quite common. This article corroborates that, despite the emotional connection driven by personal story blogs, posts focusing on statistics related to the condition may be more effective at driving readers’ preventative health intentions. This increased intention to perform preventative health action occurs regardless of the reader’s perception of risk. Further, the current research clarifies that blog posts in general, regardless of format, are more effective at driving some preventative health intentions compared with merely thinking about where one gets health information. This finding indicates that reading patient-created content may be beneficial regardless of the reader’s own risk for the health condition. Reading patient blogs may also impact perceived barriers to accessing care or perceptions of the part of our health risks that may be within our control (and not attributable to chance), leading to greater intentions to take preventative health actions. Finally, we gained insight that reading blogs, particularly those written by patients, were motivated primarily by a desire to support the patient, keep up with the patient, and learn more about the patient’s condition. Some readers did indicate that they did further research and did not take blog information at face value, but a majority reported engaging in protective health action due to their blog reading.

Although much research exists on the benefits of patients’ writing and journaling during health care treatment, the present research provides a foundation for future studies on patient and health outcomes from reading health blogs. As people seek information online about medical conditions and preventative options, patient-generated content will appear between content created by and for medical professionals; health care practitioners cannot assume patients will only read edited content from medical professionals. Understanding what type of content presentation is most effective in encouraging positive health actions may guide health care providers, patient coordinators, and patient therapists to guide recommended content styles with the greatest impact. In the case of the present studies, the use of informative statistics was the most effective in driving these intentions. This research could also inspire future studies in other health specialties to understand how these results may generalize across medical conditions and treatments.

## Appendix

Multimedia Appendix 1 Study 1 and 2 measures.

## Abbreviations

ANOVA: analysis of variance
IRB: institutional review board
MTurk: Mechanical Turk

## References

[1] Yu H, Wang Y, Wang J, Chiu Y, Qiu H, Gao M (2020). Causal effect of honorary titles on physicians' service volumes in online health communities: retrospective study. J Med Internet Res.

[2] Lepore SJ, Smyth JM (2002). The Writing Cure: How Expressive Writing Promotes Health and Emotional Well-Being.

[3] Lepore SJ, Greenberg M, Bruno M, Smyth JM, Lepore SJ, Smyth JM (2004). Expressive writing and health: self-regulation of emotion-related experience, physiology, and behavior. The Writing Cure: How Expressive Writing Promotes Health and Emotional Well-Being.

[4] Lowry F (2017). Cancer Patients Write to Heal in Unique Writing Workshop. Medscape.

[5] Miller EA, Pole A, Bateman C (2011). Variation in health blog features and elements by gender, occupation, and perspective. J Health Commun.

[6] Scheibling C, Gillett J, Brett G (2017). Making the virtual rounds: the use of blogs by health-care professionals. Journal of Communication Inquiry.

[7] Fox S, Madden M (2006). Riding the Waves of “Web 2.0”. Pew Research Center.

[8] How Many Blogs are there in 2018?. SaaS Scout.

[9] Blogging Statistics. 99Firms.

[10] Revealed: Which are the Most Popular Types of Blogs?. WPBeginner.

[11] Hesse BW, Moser RP, Rutten LJ (2010). Surveys of physicians and electronic health information. N Engl J Med.

[12] Fox S (2011). Mind the Gap: Peer-to-peer Healthcare. Pew Research Center.

[13] Moorhead SA, Hazlett DE, Harrison L, Carroll JK, Irwin A, Hoving C (2013). A new dimension of health care: systematic review of the uses, benefits, and limitations of social media for health communication. J Med Internet Res.

[14] Zhao Y, Zhang J (2017). Consumer health information seeking in social media: a literature review. Health Info Libr J.

[15] (2012). Why Most Facebook Users Get More than They Give. Pew Research Center.

[16] Chiu Y, Hsieh Y (2013). Communication online with fellow cancer patients: writing to be remembered, gain strength, and find survivors. J Health Psychol.

[17] Attai DJ, Cowher MS, Al-Hamadani M, Schoger JM, Staley AC, Landercasper J (2015). Twitter social media is an effective tool for breast cancer patient education and support: patient-reported outcomes by survey. J Med Internet Res.

[18] Thackeray R, Crookston BT, West JH (2013). Correlates of health-related social media use among adults. J Med Internet Res.

[19] Bigley IP, Leonhardt JM (2018). Extremity bias in user-generated content creation and consumption in social media. Journal of Interactive Advertising.

[20] Kotliar DM (2016). Depression narratives in blogs: a collaborative quest for coherence. Qual Health Res.

[21] McGeehin Heilferty C (2018). The search for balance: prolonged uncertainty in parent blogs of childhood cancer. J Fam Nurs.

[22] Anderson JG, Hundt E, Dean M, Keim-Malpass J, Lopez RP (2017). "The Church of Online Support": examining the use of blogs among family caregivers of persons with dementia. J Fam Nurs.

[23] Andersson Y (2019). Blogs and the art of dying: blogging with, and about, severe cancer in late modern Swedish society. Omega (Westport).

[24] Heilferty C (2009). Toward a theory of online communication in illness: concept analysis of illness blogs. J Adv Nurs.

[25] Høybye MT, Johansen C, Tjørnhøj-Thomsen T (2005). Online interaction. Effects of storytelling in an internet breast cancer support group. Psychooncology.

[26] Kraft MA (2006). The use of blogs in medical libraries. Journal of Hospital Librarianship.

[27] Sandars J (2006). Twelve tips for using blogs and wikis in medical education. Med Teach.

[28] Kim S, Chung DS (2007). Characteristics of cancer blog users. J Med Libr Assoc.

[29] Huang L, Chou Y, Lin C (2008). The influence of reading motives on the responses after reading blogs. Cyberpsychol Behav.

[30] Rapp C (2010). Aristotle's Rhetoric. The Stanford Encyclopedia of Philosophy.

[31] Cialdini R (2007). The secret impact of social norms. RSA J.

[32] Cialdini RB (2001). Harnessing the Science of Persuasion. Harvard Business Review.

[33] Resnik AJ, Cialdini RB (1986). Journal of Marketing Research.

[34] de Wit JBF, Das E, Vet R (2008). What works best: objective statistics or a personal testimonial? An assessment of the persuasive effects of different types of message evidence on risk perception. Health Psychol.

[35] liberman A, Chaiken S (2016). Defensive processing of personally relevant health messages. Pers Soc Psychol Bull.

[36] Slater MD, Rouner D (2016). Value-affirmative and value-protective processing of alcohol education messages that include statistical evidence or anecdotes. Communication Research.

[37] Loewenstein GF, Weber EU, Hsee CK, Welch N (2001). Risk as feelings. Psychol Bull.

[38] Yalch RF, Elmore-Yalch R (1984). The effect of numbers on the route to persuasion. J Consum Res.

[39] Nexøe J, Kragstrup J, Søgaard J (1999). Decision on influenza vaccination among the elderly. A questionnaire study based on the Health Belief Model and the Multidimensional Locus of Control Theory. Scand J Prim Health Care.

[40] McHugh ML (2012). Interrater reliability: the kappa statistic. Biochem Med.

[41] Taber KS (2017). The use of Cronbach’s alpha when developing and reporting research instruments in science education. Res Sci Educ.

[42] Weiss C (2020). Mayo Clinic Q and A: The importance of a skin cancer check. Mayo Clinic.

[43] Azjen I, Kuhl J, Beckmann J (1985). From Intentions to Actions: A Theory of Planned Behavior. Action Control. SSSP Springer Series in Social Psychology.

[44] Sheeran P, Conner M, Norman P (2001). Can the theory of planned behavior explain patterns of health behavior change?. Health Psychology.

[45] Conner M, Norman P, Bell R (2002). The theory of planned behavior and healthy eating. Health Psychology.

[46] Armitage CJ (2005). Can the theory of planned behavior predict the maintenance of physical activity?. Health Psychol.

[47] Klein WMP, Stefanek ME (2007). Cancer risk elicitation and communication: lessons from the psychology of risk perception. CA Cancer J Clin.

[48] What Is Cancer?. American Cancer Society.

[49] Cialdini RB (2007). Influence: The Psychology of Persuasion.

[50] Godin G, Kok G (1996). The theory of planned behavior: a review of its applications to health-related behaviors. Am J Health Promot.

[51] Shulman R (2020). Coronavirus patients turn to social media for answers, support. The Washington Post.

[52] Nobles AL, Leas EC, Althouse BM, Dredze M, Longhurst CA, Smith DM, Ayers JW (2019). Requests for diagnoses of sexually transmitted diseases on a social media platform. JAMA.

