# Supplementary material for: Impact of Medical Blog Reading and Information Presentation on Readers’ Preventative Health Intentions: Mixed Methods, Multistudy Investigation
Source: J Med Internet Res. 2021 Dec 22;23(12):e23210. doi: 10.2196/23210 (PMC8734913; doi:10.2196/23210)
Supplement: Multimedia Appendix 1 [file jmir_v23i12e23210_app1.docx]

Study 1 and 2 Measures.

| Format | Item | Type |
| --- | --- | --- |
|  |  |  |
| Open-ended | Please briefly explain your thoughts about this blog. What thoughts do you have after reading it? | Open-ended |
|  |  |  |
| Evaluation of post | Please answer the following questions about the blog post. This post was | Bipolar |
|  | Hard/Easy to understand |  |
|  | Poorly/Well written |  |
|  | Un/Highly informative |  |
|  | Un/Emotional |  |
|  | Un/Interested |  |
|  |  | Likert-type |
| Behavioral Intentions | How likely are you to see a doctor for a skin check in the next year? |  |
|  | How likely are you to monitor your skin yourself for any changes? |  |
|  | How likely are you to use sunscreen on a daily basis? |  |
|  | How likely are you to use sunscreen when going to the beach? |  |
|  | How likely are you to ask a doctor about your cancer risk? |  |
|  |  |  |
| Health Beliefs Scales | I do not want to get a skin check | Likert-type |
|  | A skin check could have unpleasant side-effects |  |
|  | In general, I am opposed to doctor visits |  |
|  | It is too much trouble for me to go to the doctor’s for a skin check |  |
|  | Doctor visits are too expensive |  |
|  | Skin checks are effective in protecting against skin cancer |  |
|  | I have an increased risk of skin cancer |  |
|  | I am concerned about the risk of getting skin cancer |  |
|  | I get sick more easily than other people my age |  |
|  | Skin cancer may lead to serious health problems |  |
|  | If I had skin cancer, I would not be able to manage daily activities |  |
|  | I am afraid skin cancer will make me very sick |  |
|  | I am very worried about getting skin cancer |  |
|  | Whenever I get sick, it seems to be serious |  |
|  | I cannot stand to get skin cancer because of my general health |  |
|  |  |  |
| Locus of Control  (Health-Related) | If I fall ill, I have the power to make myself well again | Likert-type |
|  | I myself am responsible for maintaining my health |  |
|  | If anything goes wrong with my health, it is my own fault |  |
|  | My health depends on how well I look after myself |  |
|  | When I feel ill, I know it is because I have not looked after myself well enough |  |
|  | I can pretty much stay healthy by taking good care of myself |  |
|  | If I see my doctor regularly, I am less likely to have health problems |  |
|  | The only way I can keep my health is by consulting the health care system |  |
|  | Other people have great inﬂuence on my state of health |  |
|  | The health care system keeps me healthy |  |
|  | Other people’s treatment and care decide whether I recover from illness |  |
|  | The best way for me to stay healthy is to follow doctor’s orders to the letter |  |
|  | I often feel that no matter what I do, if I am going to fall ill, it is meant to be like that |  |
|  | It seems as if my health mostly depends on sheer coincidence |  |
|  | When I am ill, I must let nature take its course |  |
|  | If I am healthy, it is because I am lucky |  |
|  | Even though I take good care of myself, I fall easily ill |  |
|  | When I fall ill, it is a matter of fate |  |
|  |  |  |
| Health Behaviors | Do you use sunscreen? | Likert-type |
|  | If you do, how often do you use sunscreen? |  |
|  | Have you ever had a skin check with a dermatologist? |  |
|  |  |  |
